# Biosorption of Microelements by *Spirulina*: Towards Technology of Mineral Feed Supplements

**DOI:** 10.1155/2014/356328

**Published:** 2014-10-19

**Authors:** Agnieszka Dmytryk, Agnieszka Saeid, Katarzyna Chojnacka

**Affiliations:** Institute of Inorganic Technology and Mineral Fertilizers, Wrocław University of Technology, Smoluchowskiego 25, 50-372 Wrocław, Poland

## Abstract

Surface characterization and metal ion adsorption properties of *Spirulina* sp. and *Spirulina maxima* were verified by various instrumental techniques. FTIR spectroscopy and potentiometric titration were used for qualitative and quantitative determination of metal ion-binding groups. Comparative FTIR spectra of natural and Cu(II)-treated biomass proved involvement of both phosphoryl and sulfone groups in metal ions sorption. The potentiometric titration data analysis provided the best fit with the model assuming the presence of three types of surface functional groups and the carboxyl group as the major binding site. The mechanism of metal ions biosorption was investigated by comparing the results from multielemental analyses by ICP-OES and SEM-EDX. Biosorption of Cu(II), Mn(II), Zn(II), and Co(II) ions by lyophilized *Spirulina* sp. was performed to determine the metal affinity relationships for single- and multicomponent systems. Obtained results showed the replacement of naturally bound ions: Na(I), K(I), or Ca(II) with sorbed metal ions in a descending order of Mn(II) > Cu(II) > Zn(II) > Co(II) for single- and Cu(II) > Mn(II) > Co(II) > Zn(II) for multicomponent systems, respectively. Surface elemental composition of natural and metal-loaded material was determined both by ICP-OES and SEM-EDX analysis, showing relatively high value of correlation coefficient between the concentration of Na(I) ions in algal biomass.

## 1. Introduction

Biosorption properties of* Spirulina* have been adapted in many techniques related with metal pollution control [1–3]. Industrial wastewater treatment or, generally, bioremediation of aquatic systems was the subject of several investigations [4–9], including smelter and refinery effluents processing [10]. Besides heavy metal ions, removal of inorganic contaminants [11, 12] and toxic organics was investigated [13], as well. Despite proven efficacy of* Spirulina* biomass,* Chlorella* species were reported as the most commonly used in current treatment technologies [14]. Nevertheless, other potential applications of* Spirulina *adsorptive capacity, such as livestock feeding and human dietary supplementation, have been already discussed in the literature [15–18]. Further studies on using this microalga as versatile biosorbent are therefore justified, since they may provide insights into both the process pathways and the mechanism of sorption.

**Figure 1:**
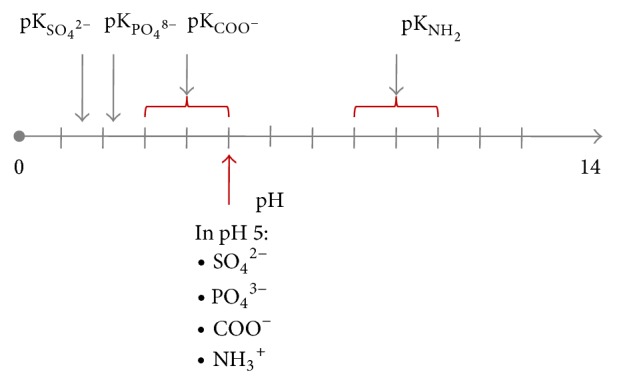
The state of ionization of functional groups on the surface of the microalgal cell wall depending on pH.

Strong molecular interactions, in particular electrostatic attractions, determine the quality of biosorbents and depend on chemical state and composition (type and quantity) of surface functional groups. The state of surface moieties, their ionization or protonation, results directly from dissociation constant (pK_*a*_ or pK_*b*_, in fact) and medium acidity (pH). Usually, value of this constant for chemical moiety in biomolecules closely corresponds to pK value of appropriate inorganic acid or base. The presence of different types of functional groups, commonly: carboxyl, phosphoryl, sulfone, hydroxyl, and amine groups on biomass surfaces, causes that the biomass has amphoteric properties. Depending on pH, acid or base character may be more or less apparent. Protonated amines are positively charged, while deprotonated are neutral. In the case of carboxyl, phosphoryl or sulfone groups, protonation neutralizes those groups, while removal of protons forms negatively charged conjugate bases [19–21]. It is especially important when concerning biomass interaction with different ions and the fact that electrical charge of biomass surface, governs sorbates adsorption. For example, the isoelectric point (pI) of microalgae* Chlorella* is about 3, while* Spirulina *pI = 2.8–3.5 [19]. In lower pH, positively charged groups (e.g., amine group) attract anions and repulse cations, while in higher pH negatively charged groups (e.g., carboxyl) act conversely. Typical changes of ionization state of functional groups on the surface of microalgae cell wall depending on pH are shown in Figure 1, along with the listing of functional groups (under the red arrow) and their forms in pH = 5, for which the highest model parameters from quantitative and kinetic examination have been obtained [20].

In present work, dried biomass of commercially available* Spirulina* sp. (lyophilisate) and cultivated under laboratory conditions* Spirulina maxima* were investigated for supplementation with microelement ions: Cu(II), Co(II), Mn(II), and Zn(II), which are important in livestock feeding. Biosorption experiments were performed in both single- and multicomponent systems, under previously evaluated conditions. Four analytical methods were used to assess the composition of natural and loaded biomass surface, as well as to verify the biosorption mechanism, in exchange, posited on the basis of earlier studies. The major binding sites were identified along with their concentrations, and thus total capacity of both examined biosorbents might be estimated. Based on changes observed in elemental composition of the* Spirulina* surface after biosorption experiments, the affinity sequence of microelement cations was determined for single- and multimetal systems separately. The applicability of microalgal biomaterials to feed supplementation was assessed due to resultant of all conducted tests.

## 2. Materials and Methods

### 2.1. Microorganisms and Media Composition

Two kinds of algal biomasses were selected for biosorption research. Commercially available* Spirulina *sp. in form of lyophilized cells was purchased from SIGMA (USA).* Spirulina maxima* was obtained from Culture Collection of Algal Laboratory (CCALA) Institute of Botany, Academy of Sciences of the Czech Republic. Microalga grew in the Schlösser liquid medium [22], composition of which is presented in Table 1.

Schlösser culture medium was prepared with analytical grade reagents and purchased from POCh S.A. (Gliwice, Poland). After cultivation, the biomass was separated by filtration, using the aquarium algae vacuum cleaner (Actizoo Zolux, France), dried at 60°C (laboratory oven, WAMED; Warsaw, Poland) for three days, and subjected to further investigation as a biosorbent.

### 2.2. Cultivation of* Spirulina maxima*

*Spirulina maxima* was cultivated in laboratory-scale reactors, at 35°C [23] for 4 months. The cultures were carried out under 12 : 12 photoperiod conditions (12 hr light : 12 hr dark cycles), provided with artificial illumination of 19 W m^−2^ intensity. In order to improve growth, algal scalability tests were performed in three consecutive systems of increasing capacity. Microalga cultivation started with a working capacity of 1 L and, due to cell passaging, 5 L and 40 L reactor systems were examined, as well.

**Figure 2:**
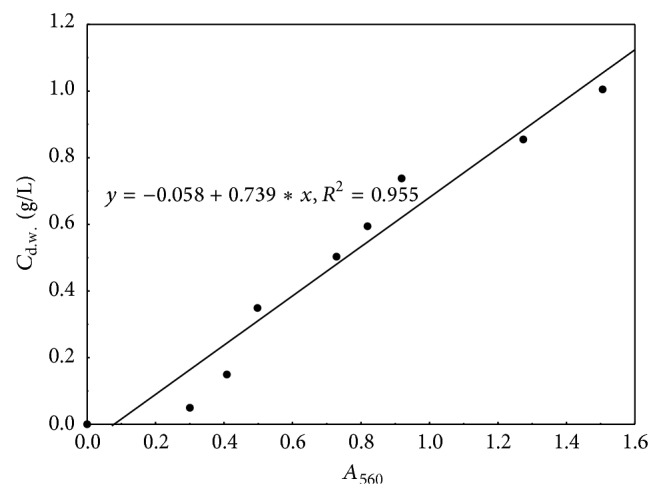
Standard curve represents the relationship between concentration of* Spirulina maxima* dry mass (*C*_d.w._) and corresponding absorbance (*A*_560_) results.

The efficiency of the* Spirulina maxima* cultivation in each of tested capacities was determined using three basic parameters: the concentration of microalgal culture, growth rate, and relative (exponential) growth rate. The first of three (*C*_*S*_) was estimated by the straight-line equation showing the correlation between concentration of microalga dry mass and absorbance measurements at 560 nm (Figure 2).

### 2.3. Semibatch Mode

**Figure 3:**
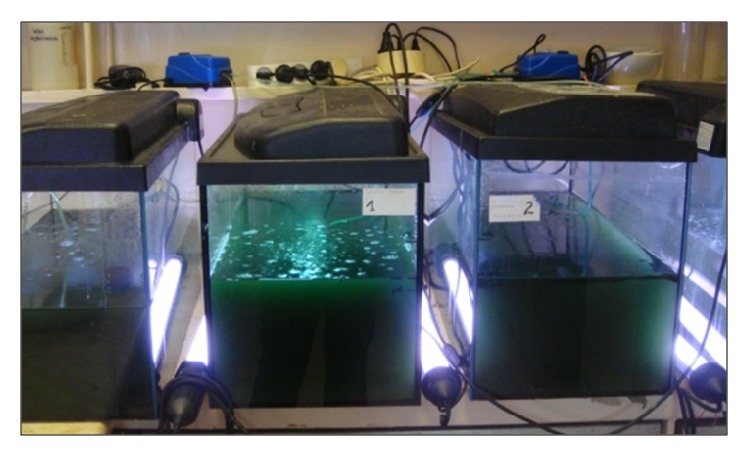
*Spirulina maxima* cultivation carried out in laboratory scale system of 40 L.

The experiment was conducted on* Spirulina maxima* culture and grown in Schlösser medium at 35°C, using 40 L-working capacity reactor equipped with two light sources (12 : 12 photoperiod) (Figure 3). The cultivation was carried out for 4 months.

During research, the systematic partial reception of microalgal biomass was performed to diminish the self-shading effect, which was verified by cell concentration measurements. The biomass was separated by vacuum filtration (Actizoo Zolux, France) seven times overall. The last separation stage gathered whole population of grown* Spirulina maxima*, in which cells after 3-day drying at 60°C (WAMED; Warsaw, Poland) were examined on biosorption properties in adequate tests described below.

### 2.4. Metal Ions Biosorption Experiments

Biosorption experiments were performed for copper(II), cobalt(II), manganese(II), and zinc(II) ions in both single- and multimetal systems, according to the procedure used in previous work [15]. Tests were carried out in a batch mode, at equal initial ion concentrations 300 mg L^−1^ at 35°C (*Spirulina* cultivation temperature) and pH 5. The microalgal biosorbent was examined at a concentration of 1 g L^−1^. The solutions of metal ions were prepared in deionized water with appropriate amounts of inorganic salt (from POCh S.A. Gliwice, Poland): Co(NO_3_)_2_ *·*5H_2_O, CuSO_4_ *·*5H_2_O, MnSO_4_ *·*5H_2_O, ZnSO_4_ *·*7H_2_O. pH of the metal ion solution was measured using Mettler Toledo Seven Multi-pH-meter (Greifensee, Switzerland) equipped with a temperature-compensating electrode InLab413. pH adjustment to the endpoint was performed with standardized NaOH or HCl solution (0.1 mol L^−1^; POCh S.A. Gliwice, Poland). The biosorption experiments were conducted in Erlenmeyer flasks containing 100 mL of Cu(II), Co(II), Mn(II), and Zn(II) synthetic solutions, shaken in thermostated water bath shaker at 150 rpm speed. The contact time of 60 minutes was evaluated for* Spirulina* biomass based on previous kinetic research [15]. After completion of each test, the microalgal suspension was separated by filtration through the filter papers, oven dried (WAMED; Warsaw, Poland), and further analyzed.

### 2.5. Fourier Transform Infrared (FTIR) Spectroscopy

FTIR spectroscopy was used to identify microalgal cell wall functional groups participating in biosorption by detection of vibration wave number changes in the sorbent surface, occurred after metal ion binding. FTIR measurements were thus performed for natural* S. maxima* dried cells and the same biomass enriched in Cu(II) (model single-metal system). In order to verify diversity of surface functional groups for different microalgal biosorbent, pristine* Spirulina *sp. lyophilisate was examined, as well. Before analyzing, a few milligrams of each alga, including Cu(II)-loaded* S. maxima*, were ground and mixed with potassium bromide disks in an amount provided the biomaterial concentration of 2%, by mass. The FTIR spectra of prepared samples were collected by the use of a Perkin-Elmer System 2000 (Waltham, MA, USA) equipped with deuterated triglycine sulfate (DTGS) and mercury cadmium telluride (MCT) detector. The assay was conducted within the wave number range of 400–4000 cm^−1^ (midinfrared region) using a potassium bromide window, at room temperature (26 ± 1°C) [24]. FTIR spectra were elaborated with a System 2000-compatible software (Perkin-Elmer; Waltham, MA, USA), by use of which background calculation was performed to set necessary entries for result assessment. Obtained background, corresponding to pure KBr, was automatically subtracted from each sample spectrum. All spectra were plotted using the same scale on the transmittance axis.

### 2.6. Potentiometric Titration of the Biomass

Potentiometric titration was performed to more specifically identify ion-binding groups, compared to FTIR spectroscopy, and to determine their concentrations onto microalgal cell wall. Measurements were conducted with both microalgal biomasses, according to the method elaborated in previous works [20, 24]. Biomass samples of 0.2 g suspended in deionized water (200 mL) were titrated with 0.1 M NaOH till pH 11.5 and reversely with 0.1 M HCl till pH 2.5, both solutions were standardized (POCh S.A., Gliwice, Poland). The pH change was monitored after single dose of titrant, when the record stabilization was achieved. As control (blank) sample, deionized water was used. Before each titration test, the water was purged of dissolved CO_2_ by 3-hour bubbling with argon.

### 2.7. Scanning Electron Microscopy with Energy Dispersive X-Ray (SEM-EDX) Analytical System

SEM-EDX analysis was carried out at Wrocław University of Environmental and Life Sciences (Electron Microscope Laboratory) to investigate the biosorption effect on element content of biomass cell wall, including both micro- and macroelements, and map the distribution of ions bound to its surface, as it was reported by Michalak and coworkers [25]. Tests were conducted using pristine and ion-enriched cells of lyophilized* Spirulina* sp., loaded with Co(II), Cu(II), Mn(II), and Zn(II) cations in both single- and multimetal system. Microalgal samples, a total of six, were fixed in 2.5% of glutaraldehyde (Sigma, http://www.sigmaaldrich.com/) and next dehydrated by ethanol (from 30% till 100% concentration). Treated biomass were sampled and prepared in two planes, enabling to observe cross-section and its surface. All samples were mounted on proper stub and gold sputtered thereafter using HHV Scancoat Six equipment (Crawley, Oxfordshire, UK). Observation and photographs of microalgae surface composition were taken with a Leo Zeiss 435 VP SEM scanning electron microscope (Oberkochen, Germany), operating at 20 kV. An additional equipment of the microscope was a RÖNTEC GmbH energy dispersive X-ray system (Berlin, Germany), by which the concentration of particular cell wall constituent was recorded. As a result, the X-ray spectra of elemental composition, specific for natural and metal ion-enriched* Spirulina* sp. surface were obtained.

### 2.8. Inductively Coupled Plasma-Optical Emission Spectrometry (ICP-OES)

ICP-OES technique was used to determine and compare total content of the elements in microalgal biosorbent before and after metal ion binding. This analysis was proven to be precise and appropriate in evaluation of biosorption efficacy, considering applicability to feed supplementation in particular [26]. In conducted research, ICP-OES was performed as a complementary analysis to SEM-EDX system, intended for verification of the scanning electron microscopy usefulness to record and elaborate data from biosorption experiments with* Spirulina*. Measurements were carried out on* Spirulina* sp. samples with and without metal ion enrichment, using VISTA-MPX spectrometer (Varian; Victoria, Australia) with ultrasonic nebulizer in the Chemical Laboratory of Multielemental Analyses at Wrocław University of Technology accredited by ILAC-MRA (Varian VISTA-MPX ICP-OES) and the Polish Centre for Accreditation (number AB 696). According to formerly elaborated procedure [15], microalgal samples of c.a. 0.5 g suspended in 5 mL of 65% nitric acid Suprapur from EMD Millipore (Merck KGaA; Darmstadt, Germany) were subjected to pressure microwave digestion (mineralization) in Teflon vessels using microwave oven Milestone MLS-1200 (Sorisole, Bergamo, Italy). After mineralization, all samples were diluted to 50 mL and the concentrations of metal ions were detected spectrometrically (VISTA-MPX, Australia) in triplicate. The apparatus was calibrated with standard solutions (1.0, 10, 50, and 100 mg L^−1^), prepared based on the 100 mg L^−1^ multielemental standard Astasol (Prague, Czech Republic). Optimization of the test parameters was performed as described before [26]. The analytical process of digested biomass samples was controlled by the use of Polish Certified Reference Material for multielement trace analysis, Oriental Tobacco Leaves (CTAOTL-1), obtained from the Institute of Nuclear Chemistry and Technology (Poland, http://www.ichtj.waw.pl/drupal/) [26].

## 3. Results and Discussion

### 3.1. Reactor Capacity Selection for* Spirulina maxima* Cultivation

Three comparative experiments in 1 L-, 5 L-, and 40 L-capacity reactor were performed to select the working scale, in which* Spirulina maxima* would be cultivated most effectively. The biomass yield was verified by comparison of growth rate and relative growth rate results, obtained for each cultivation.

**Figure 4:**
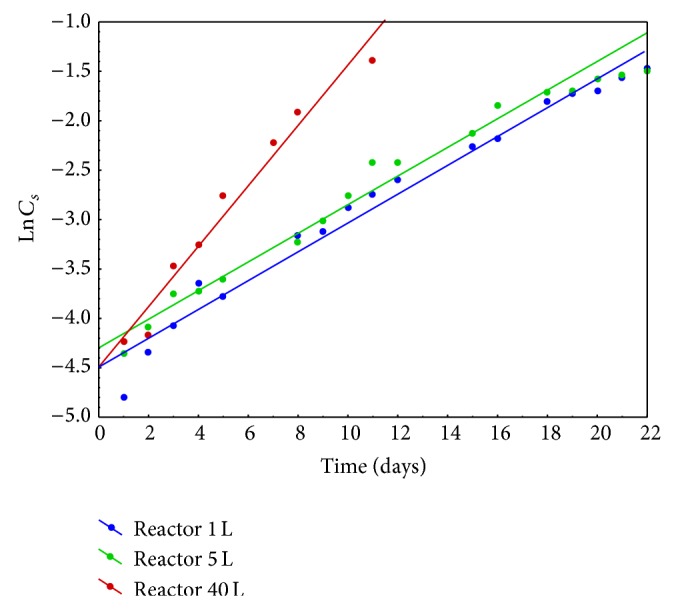
Correlation between* Spirulina maxima* concentration, expressed as natural logarithm and cultivation time for three tested systems.

Relative growth rate (*μ*) is an experimental value, specific to particular microbial system. *μ* is proportional to biomass concentration increase during the logarithmic growth phase over a certain time period, which is expressed by

(1) $$\begin{array}{c} \mu =\frac{\ln C_{S}^{t}-\ln C_{S}^{0}}{t},\\ \left[\mu \right]\text{=}\left[{\text{day}}^{-1}\right], \end{array}$$

where *t*: time period, after which the culture concentration was measured (assuming *t* ^0^ = 0), *C*_*S*_^*t*^: the culture concentration after time *t*, and *C*_*S*_^0^: the initial concentration of the culture. Relative growth rate is usually determined from the graphically depicted correlation of ln⁡⁡*C*_*S*_ = *f*(*t*) (Figure 4). The linear regression is described by

(2) $$\begin{array}{c} \ln C_{S}^{t}=\mu \cdot t+\ln C_{S}^{0}, \end{array}$$

and parameter *μ* is the slope.

Results of relative growth rate obtained for* Spirulina maxima *cultures that grew in reactors with all selected capacities were presented in Table 2.

Full characterization of the microbial cultivation required evaluation of biomass growth rate (*r*_*x*_), as well. This parameter is expressed by proportional relationship between relative growth rate and the culture concentration in exact time of measurement as follows:

(3) $$\begin{array}{c} r_{x}=\mu \cdot C_{S}^{t},\\ \left[r_{x}\right]=\left[\text{g}\cdot {\text{L}}^{-1}\cdot {\text{day}}^{-1}\right]. \end{array}$$

**Figure 5:**
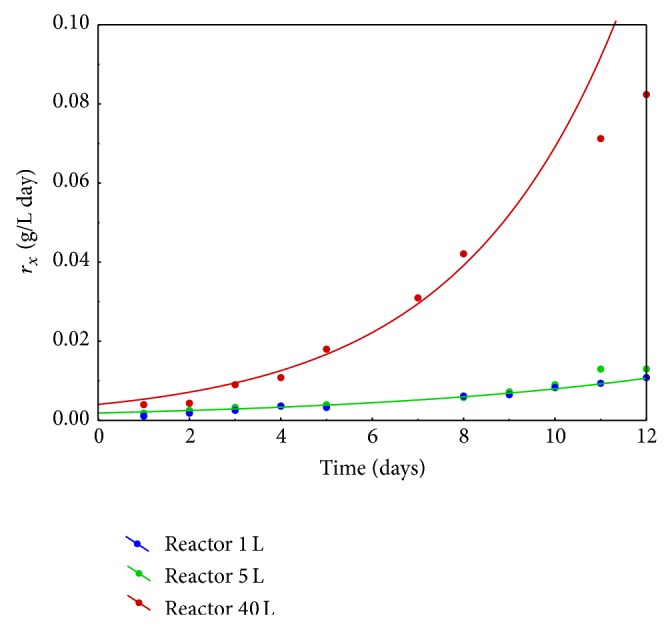
Comparison of* Spirulina maxima* growth rates for cultures in different capacities during two first weeks of cultivation.

Changes in values of growth rate during two first weeks of the research, including each examined system, are depicted in Figure 5.

Considering obtained results of growth rate and relative growth rate, the explicit correlation between reactor capacity and cultivation yield was noted. The lower the capacity system was used, the lower the rate of microalga growth was achieved. Such disparities were likely caused by self-shading effect. The highest of tested capacities was confirmed to be, simultaneously, the most effective. Thus, it was decided to cultivate* Spirulina maxima* cultures, destined for biosorption experiments, in 40 L reactor working volume in semibatch mode (Section 3.2).

### 3.2. Semibatch Mode in 40 L Reactor System

**Figure 6:**
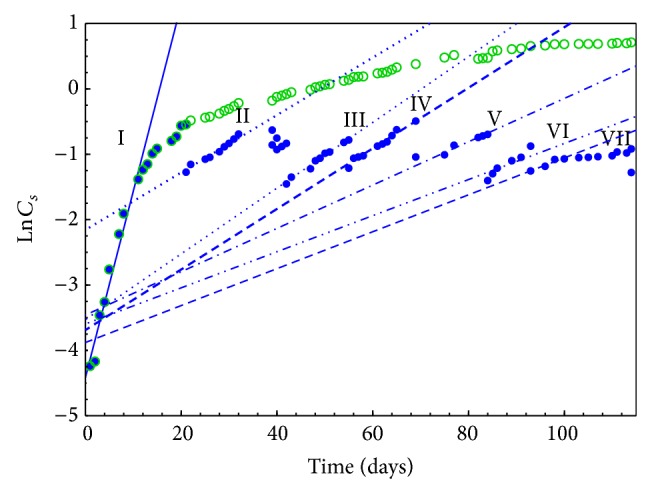
Kinetics of* Spirulina maxima* growth in semibatch culture.

Semibatch mode was used to optimize* Spirulina maxima* growth during 4-month cultivation. Except the biomass separation at the end of test, microalga cells were filtered out in the meantime, when stabilization of their concentration was noted (lag phase beginning). The next day after each of six partial receptions performed, a renewed increase in the biomass concentration was recorded (Figure 6).

Due to performing cultivation in semibatch mode, seven distinctive relative growth rates were evaluated, summary of which is presented in Table 3.

The subsequent biomass separations resulted in *μ* decrease. It was noticed that average of the relative rate values II–VII (0.0382 day^−1^) was 87% lower compared with the rate achieved at the beginning of the culture (0.285 day^−1^). Limiting the relative growth rate was likely related to medium alkalization, caused by the microbial consumption of CO_2_, released as a product of medium constituent decomposition (HCO_3_^−^⇄CO_2_ + OH^−^).

Due to similarity of the rate results obtained after fifth and sixth exceeded biomass filtration (*μ* numbers VI and VII), further cultivation was discontinued and followed with biosorption research using, previously dried, microalga cells.

### 3.3. Fourier Transform Infrared Spectroscopy

FTIR analysis was confirmed as efficacy method for identification of both total and metal ion-binding groups occurred on the biosorbent surface [27]. Evaluation of the adsorptive properties of biological materials, including algae, with FTIR spectra was investigated in a number of reports. Many studies focused on applicability of seaweeds to aqueous media treatment by removing heavy metal ions, such as Pb(II), Cd(II), Ni(II), U(VI), and Th(IV) [28–30]. There are also some works, in which algal biomass is subjected to interactions with cations of nutritional value [24, 31]. However, no literature data was found to provide information on usability of Fourier transform infrared spectroscopy in evaluation of enrichment* Spirulina* species with microelements.

**Figure 7:**
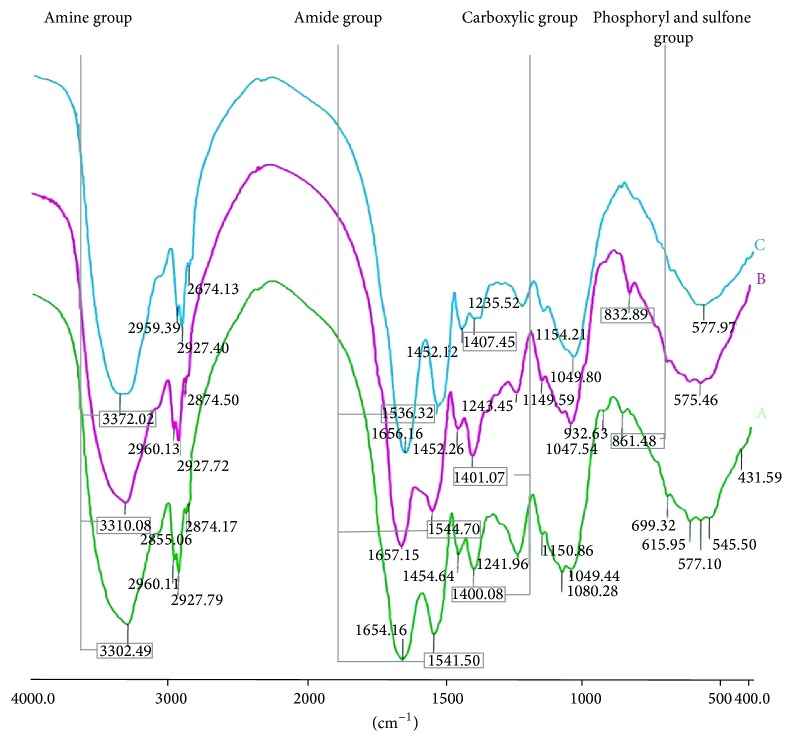
Normalized FTIR absorption spectrum of selected functional groups exposed on cell wall surface of microalgae biomass. A (green line) pristine* Spirulina *sp., B (pink line) pristine* Spirulina maxima* and C (blue line) Cu(II)-enriched* Spirulina maxima*.

Results of FTIR analysis (Figure 7) proved the variety of functional groups found on the cell wall surface of examined biosorbents. Each group in its free form, that is, without metal ion loaded, was detected at strictly defined wave number, giving a specific signal (band). A set of all bands spectrum, which depicts the full surface composition of particular biomass is named as IR “fingerprint” [32].

Figure 7 shows the bands representing the specific groups detected on the cell wall of pristine and Cu(II)-loaded* Spirulina maxima *(Figures 7(B) and 7(C), resp.), as well as lyophilized* Spirulina *sp. (Figure 7(A)). Spectra of both natural microalgal biomasses proved their great similarity in the composition of surface groups. At the same time, small differences in the wave number, at which particular band was noted, confirmed the efficacy of FTIR analysis in identifying samples of biological material. Run sequence plot of ion-enriched microalga differed, at some points, from pristine microalgae results due to interactions of cell wall groups with metal cations, which caused visible signal changes.

Considering spectra of natural biosorbent surface, a few functional groups might be identified. At the highest wave number of 3,300–3,310 cm^−1^ amine bands were detected. Stretching bands at 1,650 cm^−1^ (asymmetrical) and 1,400 cm^−1^ (symmetrical) corresponded to carbonyl groups (C=O) of protein primary amides and carboxylate ions, respectively. The band at 1,540 cm^−1^ represented the stretching vibrations of protein secondary amides (–NH). The band at 1,240 cm^−1^ was given by free –CO bound, while stretching vibrations at 1,150 and 1,050 cm^−1^ corresponded to CC/CO mode of polysaccharides ethers. Within a wave number range of 1,240–1,050 cm^−1^ hydroxyl bands were detected, as well. The detailed summary of all bands observed on the microalgae spectra was shown in Table 4 [33, 34].

The group(s) primarily concerned with metal ion interaction might be indicated with differences in vibration intensity (transmittance) or band wave number, noted between treated and nontreated biomass spectra. FTIR records for Cu(II)-loaded biomaterial showed the characteristic peak shifting of amine group, which occurred at 60 units higher wave number, compared to nontreated microalgal cells. A small change in peak positions of secondary amide (lower wave number) and carboxyl (higher wave number) groups was also observed; however, obtained differences were not as evident as in the case of amine group. Thus, verification of significance of both amide and carboxyl groups in ion sorption was required. Nevertheless, the band intensities of amine, amide, and carboxyl groups present on Cu(II)-*S. maxima* surface were higher than for pristine microalga. Such results confirmed ionic interaction between these groups and metal cations, which was in accordance with the published literature [21].

On the other hand, affecting ion sorption by hydroxyl group, commonly considered as one of the major binding site on the biomass surface [21], could not be evaluated, since none of the obtained records represented this group only. There was a peak shift observed for all bands of Cu(II) loaded microalga, which were recognized as –OH bound vibration positions (1,235.52, 1,154.21, and 1,049.80 cm^−1^). Therefore, it was assumed that hydroxyl group interacted with metal ions and further investigation was performed to prove this statement (Section 3.4).

In case of Cu(II)-*Spirulina maxima* spectrum, no band corresponding to neither phosphoryl (*≣*P–O–) nor sulfone (in deprotonated form of sulfonate, –SO_3_–) groups was detected, as well. On the other hand, these valleys were detected for pristine* Spirulina* sp. and* S. maxima* at 862 and 830 cm^−1^, respectively. Since occurring of particular band depended on whether biomass was subjected to biosorption or not, surface group represented by this band should be involved in ion binding. Thus, it was concluded that metal cations were likely bound to* Spirulina maxima* biomass with phosphoryl and deprotonated sulfone, occurring mainly in sulfonated polysaccharides, group [35].

FTIR studies revealed that the surface composition of* Spirulina *sp. is similar to* Spirulina maxima *and thus comparable adsorptive properties would be expected. As a consequence, the same functional groups were considered to be affected by the presence of metal ions and responsible for their sorption.

The spectrophotometric records were confronted with potentiometric titration in order to complement the surface characteristics of* Spirulina* biomass, concerning both type and capacity of the major binding sites.

### 3.4. Potentiometric Titration of Biosorbent Surface

The validity of using the potentiometric titration to investigate the nature of biomass surface is based on a postulate that ion exchange between biomass binding sites and metal cation solution is the dominant mechanism of biosorption. The major functional groups involved in cation binding in the pH range 2–12 were identified, as well [20, 24]. In conducted research, potentiometric titration was performed to verify the results of FTIR analysis and provide detailed information on ion binding sites of* Spirulina* sp. and* Spirulina maxima* cell walls.

The experimental data from potentiometric titration were analyzed with three models, assuming the presence of one, two, and three types of functional groups on biomaterial surface, respectively [20]. Since the algal biomass has been shown to act as weakly acidic ion exchanger, the model of multiprotic acid might be used to characterize its titration curve. In order to improve model conformance with experimental conditions, modification considering different concentration of each surface functional group was introduced [20, 24]. Elaborated model was expressed by a nonlinear correlation between molar ratio of added titrant (*x*_add_) and pH value, described with one of three equations presented in Michalak and Chojnacka report [24]. Evaluation of fitting particular model to the experimental results required estimating acidic dissociation constant (*K*_*a*_) values and heights of jumps (drop in *x*_add_) observed in the titration curve, which were determined based on nonlinear regression and the first and the second derivative course using Mathematica v. 3.0 software (Wolfram; Hanborough, Oxfordshire, United Kingdom) [20, 24]. In accordance with previous studies [15, 19, 20, 24, 25], the model assuming three different types of ion binding groups was the best fitted to the results from* Spirulina *sp. and* Spirulina maxima *potentiometric titration. Thus modified model for triprotic acid was used to describe experimental data (see (4)) [20]:

(4) Xadd=a1+10−pH/Ka2+10−2pH/(Ka1·Ka2)+10pH·Ka3 +2·b1+10−pH/Ka1+10pH·Ka2+102·pH·Ka2·Ka3 +(3·c)(1+10pH·Ka1+102·pH·Ka1·Ka2 +103·pH·Ka1·Ka2·Ka3)−1.

Model parameters* a*,* b*, and* c* equate to heights of subsequent jumps in the regression curve and represent molar ratios of 1st, 2nd, and 3rd identified functional group, respectively. According to the definition, the sum of molar ratios determined for individual components of the solution is always 1; thus, in proposed model above *a* + *b* + *c*  =  1. At the same time, the sum of jumps heights is equal to molar ratio of added titrant (*a* + *b* + *c*  =  *x*_add_). Considering both correlations, the solution of (4) is *x*_add_ = 1.

**Figure 8:**
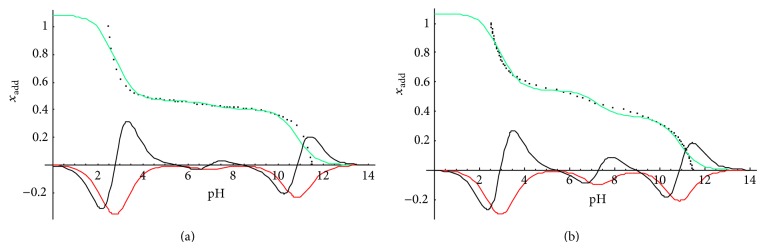
Potentiometric titration results of the* Spirulina maxima *(a) and* Spirulina* sp. (b) biomass samples. Green curve represents model curve describing the experimental data, red curve, the first derivative of the model curve, and black curve, the second derivative of the model curve.

The use of accepted model for a triprotic acid to describe potentiometric titration records gave a curve with three flex points (equivalence points of titration) [24], which was shown in Figure 8 along with the first and the second derivative course.

Acidity constants of binding sites on microalgae surface were evaluated from logarithmic measures, that is, pK_*a*_, which corresponded to zeros of the second derivative curve, calculated from the consecutive titration equivalence points [24]. Therefore, pK_*a*_ might be considered as specific pH value. In fact, pK_*a*_ determines such pH, above which functional group of particular type are in the ionized form, mostly, and hence capable of exchanging H^+^ with metal cations from the solution [20]. On the basis of determined pK_*a*_ values (black curve in Figure 8, Table 5), carboxyl, phosphoryl (phosphate, in particular) and either amine or hydroxyl groups [36–39] were identified as ion binding sites present on both* Spirulina *sp. and* S. maxima* cell walls. Obtained results were partially consentient with those from Fourier transform infrared analysis, besides –OH, significance of which was not evident on IR spectra (Figure 7). The disparity between these two evaluations might be caused by conducting ion exchange at different pH values. Biosorption experiments preceding spectrophotometric assay were carried out at pH 5, at which hydroxyl groups were not involved in cation binding due to their protonated form [20]. During potentiometric titration, solution pH exceeded pK_*a*_ enabling –OH to metal ion sorption.

On the other hand, potentiometric titration did not confirm involvement of sulfone group (as –SO_3_–) in cation exchange, while FTIR spectrometry proved otherwise. It was likely resulted from low sulfonate pK_*a*_ value, determined in the range of 1.0–2.5 [40], which was below the lowest pK_*a*_ estimated from the titration curve—pK_*a*_ = 2.66, assigned to carboxyl group. Since interaction of metal ions with deprotonated sulfone groups was evident on FTIR spectra, it might be concluded that –SO_3_– occurred on the microalgal surface at insufficiently high concentration to be considered as the major binding site.

Based on FTIR records, capability of amide group to sorb cations was taken into consideration, as well. However, as opposed to –SO_3_–, neither peak shifting nor band intensity increase, noted after biosorption experiments, provided explicit information on interaction between surface amids groups and metal cations. Thus, involvement of –NH (2° amides) in ion exchange was verified with potentiometric titration by comparing amide pK_*a*_ to those evaluated based on model (4). None of obtained results corresponded to amides (pK_*a*_ = 10.0) [41] and the highest value of determined experimentally pK_*a*_ was assigned to either amine or hydroxyl group [15, 20]. Hence, it was claimed that amide groups did not act as binding sites or their capability to bind cations was not significant enough to be confirmed.

The concentrations of surface functional groups of each type were estimated from adequate molar ratio (height of jump) and cation exchange capacity (*q*_cat.  exch._) of the* Spirulina* biomass at the investigated pH range as follows:

(5) $$\begin{array}{c} q_{\text{cat}.\;\text{exch}.}=\frac{{\text{mEq}}_{\text{biosorb}.\;\text{susp}.}^{\text{titrant}}-{\text{mEq}}_{\text{blank}}^{\text{titrant}}}{m_{\text{biosorbent}}},\\ \left[q_{\text{cat}.\;\text{exch}.}\right]=\left[\text{meq}\cdot {\text{g}}^{-1}\right]. \end{array}$$

Cation exchange capacity is an experimentally evaluated parameter, which defines capability of solid phase (sorbent) treated with an ion solution to replace surface-bound ion by another [42]. In practice, *q*_cat.  exch._ is proportional to the quantity of standardized solution (acid or base) added per unit of biosorbent mass, during entire titration [24]. The use of titrant was calculated from a difference between amounts consumed by biomass suspension and blank sample (pure water), expressed in units of milliequivalance ([mEq_sample_^titrant^] = [meq]).

There were three jumps noted in both potentiometric titration curves obtained in the test (green lines in Figure 8). Each of jumps corresponded to specific functional group, pK_*a*_ value of which equated to flex point occurred in the jump line [24]. Hence, the concentration of particular binding site, *C*_carboxyl_, *C*_phosphoryl_, *C*_amine/hydroxyl_, exposed on* Spirulina* cell surface was determined with equations (6), (7), and (8), respectively:

(6) $$\begin{array}{c} C_{\text{carboxyl}}=a\cdot q_{\text{cat}.\;\text{exch}.}, \end{array}$$

(7) $$\begin{array}{c} C_{\text{phosphoryl}}=b\cdot q_{\text{cat}.\;\text{exch}.}, \end{array}$$

(8) $$\begin{array}{c} C_{\text{amine}/\text{hydroxyl}}=c\cdot q_{\text{cat}.\;\text{exch}.}, \end{array}$$

(9) $$\begin{array}{c} \left[C_{\text{functional}\;\text{group}}\right]=\left[\text{meq}\cdot {\text{g}}^{-1}\right]. \end{array}$$

The results of the functional group concentrations, as well as total content of identified binding sites, for both* Spirulina *biomasses were shown in Table 5.

Despite the same type of groups capable of cation exchange, microalgae biosorbents differed in the incidence of binding sites on the cell wall.* Spirulina maxima* surface was characterized by 3.0 and 3.7 times higher content of both –COO and *≣*P–O–, and –NH_3_/–OH, respectively, compared to* Spirulina* sp. Literature reported that surface composition and, thus, adsorptive properties of biomass depend on its species and condition of growth [20]. Previous studies on* Spirulina* sp. in four different morphological types proved that environmental condition manipulating affected the quality of biosorbent and, as a result, the efficacy of ion exchange [20]. Comparing two biosorbents in terms of surface composition, only, identical treatment during whole investigation is required. Since no detailed information was known on* Spirulina* sp. cultivation, conducted tests could not be considered as comparable. The aim of our own experiments was to confirm the applicability of both microalgae to microelement sorption, as well as to verify whether proposed conditions of culture growth provided material which is equally good with commercial product. Obtained results showed that* Spirulina maxima* grown under laboratory conditions would be more efficient biosorbent than lyophilized* Spirulina* sp. purchased on a market, due to a higher number of binding sites.

Evident disparities in the content of surface functional groups were proved; however, a few similarities between* Spirulina* sp. and* S. maxima* records were observed, as well. In case of both microalgae, the determined concentrations of carboxyl and either amine or hydroxyl groups were 1,5–1,9 times higher compared to the content of phosphoryl group. Such results might be caused by a different amount of compounds, from which these groups were derived. Since algal cell wall is built with polysaccharides, at most [43], diversity of these components influences the structure of binding sites exposed on the biomass surface. Hence, obtained composition of surface functional groups is specific for particular biosorbent species, which is in accordance with “fingerprint” theory of FTIR spectra. Similarity of the concentration ratios between each pair of identified groups ([–COO] : [*≣*P–O–], [–COO] : [–NH_3_] or [–OH], [*≣*P–O–] : [–NH_3_] or [–OH]) was likely resulted from close morphological resemblance between examined algae.

Although on* S. maxima* surface either amine or hydroxyl groups were mostly exposed, the carboxyl groups were considered to act as the major binding sites of both microalga. As carboxyl groups had the pK_*a*_ value of a little over 2.5 (the endpoint of titration), they were in the form of carboxylate ions capable of cation exchange within almost whole range of pH, at which potentiometric titration was performed [20]. Moreover, deprotonation of binding sites, which made them available to metal ions, was enhanced along with pH increase. In case of titration curve, it might be concluded that higher pH improved the effectiveness of cation exchange.

### 3.5. SEM-EDX Analysis of Algal Biomass Surface Enriched in Microelements

Previously conducted analysis enabled characterization of both type and capacity of binding sites present on* Spirulina* cell wall. However, neither FTIR spectroscopy nor potentiometric titration provided information on changes in elemental composition of microalgae surface, resulted from metal ion biosorption. Therefore, scanning electron microscopy with an energy dispersive X-ray analytical system technique was carried out to compare the concentration of elements on natural and cation-loaded microalgae cell wall. Since the same functional groups involved in ion exchange were proved to occur on both* Spirulina* sp. and* S. maxima* surface, it was decided to perform SEM-EDX assay with one of them only. For further, multi-elemental analysis lyophilized* Spirulina* sp. was selected. Although ion exchange capacity of* S. maxima *was proved to be over 3 times higher, commercial microalgae had certain quality, which provided reliable records. Moreover,* Spirulina* sp. was used as biosorbent in previous tests for exploring the biosorption mechanism [15, 20] and thus comparison of the results could be made.

SEM-EDX analysis was used in several studies to evaluate sorption of microelement cations onto seaweeds biomass [25, 27]. Since studies on macroalgae proved this technique as suitable for investigating feed supplementation via biosorption, it was decided to apply SEM-EDX analytical system in present work.

**Figure 9:**
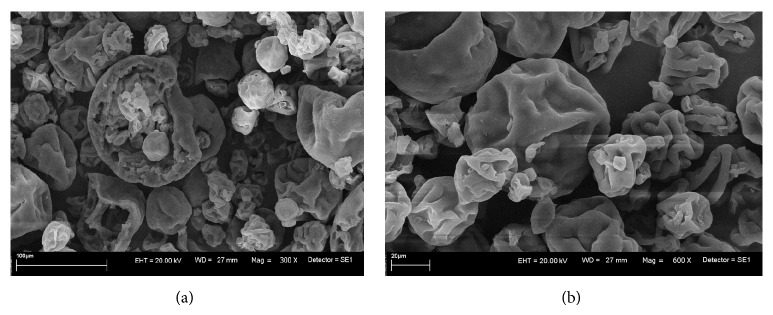
Images of lyophilized* Spirulina *sp. obtained with scanning electron microscope (SEM).

The assay was preceded by biosorption experiments, performed in both single- and multimetal (Co^2+^, Cu^2+^, Mn^2+^, Zn^2+^) system and under the specified conditions (Section 2.4). The surface composition of the microalgae samples loaded with each metal ion, in total of five, and all cations at once were analyzed and compared to the results for natural* Spirulina* sp. The photographs of the surface structure of pristine* Spirulina *sp. lyophilisate were taken with scanning electron microscopy and shown in Figure 9.

**Figure 10:**
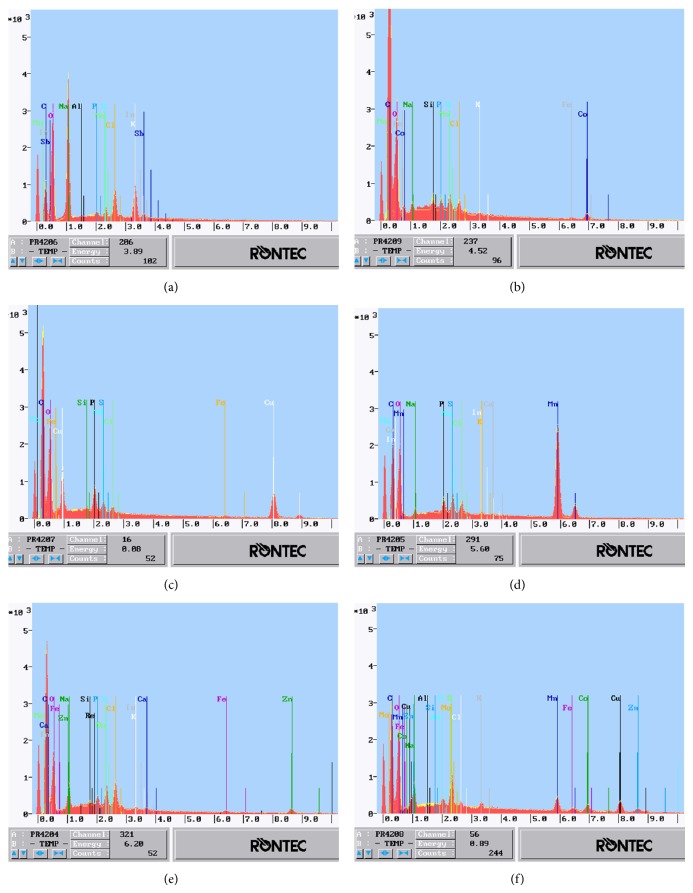
X-ray spectrum, mineral trace analysis of* Spirulina *sp. surface. (a) pristine microalgae biomass;* Spirulina *sp. enriched in (b) Co(II) ions, (c) Cu(II) ions, (d) Mn(II) ions, and (e) Zn(II) ions; (f)* Spirulina* sp. enriched in all Co(II), Cu(II), Mn(II), and Zn(II) ions.

The samples of treated and nontreated biomass were subjected to elemental analysis using EDX system and six distinct X-ray spectra were obtained (Figure 10).

The results obtained from EDX analysis showed that each of examined* Spirulina* samples gave different set of bands due to changes in the surface composition, caused by biomass treatment with cation(s) in single- or multimetal system. On the spectra of microalgae enriched in Co(II), Cu(II), Mn(II), Zn(II) ions, and all of them together, the bands representing overbalanced concentration of these cations were observed in Figures 10(b)–10(f), respectively, while there were no corresponding bands recorded on the natural biomass spectrum (Figure 10(a)). Diversity of the cell wall composition among loaded* Spirulina* samples was noted, as well. Previous studies [20] proved that efficiency, with which surface functional groups bound ions varied depending on particular element (metal). The affinity of biosorbent for sorbate is specific and attributable to both the type and valence of ion and pK_*a*_ value of the binding functional group. Thus, this parameter needs to be separately verified for each system. In present research, a relative affinity between* Spirulina* sp. and Co(II), Cu(II), Mn(II), and Zn(II) ions was determined based on SEM-EDX results, by comparing the concentration of the one type of bound ion to other records. The summary of element concentration evaluated for pristine* Spirulina* and samples after biosorption experiments was presented in Table 6.

The results obtained during X-ray exams on cation-loaded* Spirulina* sp. showed increased content of all microelement ions, in which biomass was enriched via biosorption. For single-metal system, the concentration of Co(II), Cu(II), Mn(II), and Zn(II) was about 12, 71.5, 1,380, and over 1,760 times higher, respectively, compared to natural microalgae, while experiment in multicomponent system gave an increase of the same cations at 14.5, 22, 87, and over 180 times. (The increase of zinc ion concentration was determined as the ratio of the corresponding result for Zn(II)-enriched* Spirulina* sp., in either single-or multimetal system, to the lower detection limit of EDX system.) Although, performing biosorption in multimetal system was less effective, it provided supplementation with all examined ions, at once. Thus, making a decision concerning more preferable method of enrichment* Spirulina* with cation(s) depends on the application, to which the loaded algal biomass is intended.

Analysis of the surface composition of various elements justified the relevance to perform parallel biosorption experiments in single- and multimetal system, since the presence of competing cations significantly affected the binding preferences of biomass. Disparities in absolute concentration of each microelement and the efficacy of its interacting with surface functional groups, compared to other elements, were noted. The* Spirulina *sp. affinity for examined metal ions in single- and multicomponent system fallowed Mn(II) > Cu(II) > Zn(II) > Co(II) and Cu(II) > Mn(II) > Co(II) > Zn(II), respectively. The concentration difference between the most and the least easily bound cation was 10 times higher in case of samples prepared in single-metal system than in the presence of competing ions. As all cations were equally charged and biosorption experiments were carried out using the same biosorbent species, it was concluded that interaction, and hence affinity, between biomass of* Spirulina* sp. and each microelement cation depended on sorbate type(s), only.

Apart from increased concentration of cobalt, copper, manganese, and/or zinc, the surface composition of loaded microalgae showed significantly lower content of alkali metal compared to natural cells. Such results might be referred to as ion exchange mechanism, via which biosorption primarily occurs [35, 44]. The difference of sodium concentration before and after biomass enrichment was more evident than in the case of potassium; thus Na(I) was claimed to be most involved in ion exchanging among cations naturally bound with surface functional groups. Both sodium and potassium ions interacted with all examined sorbates. According to the literature, alkaline earth metal ions might also participate in biosorption [35, 44], which was confirmed in present work, since the concentration of calcium cations decreased due to loading* Spirulina* sp. with microelement(s). However, as opposed to Na(I) and K(I), exchanging Ca(II) with other metal ions was noted in the presence of Co(II) and Cu(II) cations and in multicomponent system, only.

The composition of microalgae surface, besides ions exchanged during biosorption, contained elements, such as cell wall building blocks (e.g., carbon) or functional group constituents (e.g., phosphorus), the concentration of which should not have varied after microelement ion binding, while it did. Disparities in records for natural and enriched* Spirulina* sp. were likely observed due to conformational changes of neighboring functional groups, as they interacted with different cations. According to posited mechanism of biosorption-ion exchange, cation binding onto biosorbent occurs through electron donor-acceptor interactions, resulting in a replacement of naturally bound ions: Na(I), K(I), and/or Ca(II). Both alkali and alkaline earth metal ions are larger in size compared to microelement cations used for biosorption experiments. The ionic radius for Na(I), K(I), and Ca(II) is 95 pm, 133 pm, and 99 pm, respectively; in contrast, ionic radius for Co(II), Cu(II), Mn(II), and Zn(II) is in the range of 73–80 pm [45, 46]. The exchange of larger cations to smaller cations affects the spatial distribution of ions onto the biosorbent surface and either revealing or concealing the particular elements on* Spirulina *sp. surface might be observed as increase or decrease of the corresponding bands in the X-ray spectra.

### 3.6. Comparison of SEM-EDX and ICP-OES Techniques

**Figure 11:**
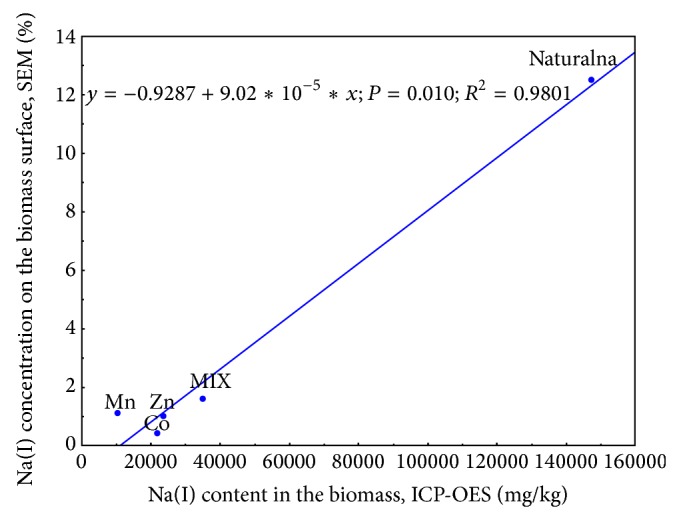
Correlation between the concentration of Na(I) ions on* Spirulina *sp. surface, assessed by ICP-OES and SEM-EDX analysis. Naturalna—pristine microalgae; Co, Mn, Zn—biomass enriched in Co(II), Mn(II) and Zn(II) cations, respectively; MIX—biomass enriched in metal cations in multi-component system. Values on the *x* and *y* axis indicate Na(I) concentration determined by ICP-OES and EDX and analytical system, respectively.

Inductively coupled plasma-optical emission spectrometry was used in several studies as the suitable method for investigating the elemental composition of biosorbent surface, thus assessing the mechanism of the process and applicability of the biomass to binding particular ions [25, 26]. Since ICP-OES gave reliable and explicit results from previous experiments with* Spirulina* [15–17, 20], it was decided to employ this technique to verify the records from SEM-EDX analysis, in present work. However, there was no cation exchanged during biosorption, the atomic concentration of which was evaluated in all examined samples. Thus, the correlation coefficient between ICP-OES and SEM-EDX analytical techniques was assessed on the example of the results obtained for sodium ion, as it was most frequently replaced by microelement cations regardless of the system tested. The ratios of Na(I) concentration on natural and loaded* Spirulina* surface, determined by both compared analysis, were depicted graphically and linear regression equation was determined (Figure 11). Records for microalga loaded with Cu(II) were not involved in estimating the ICP-OEC/SEM-EDX correlation coefficient, since the content of sodium ions was under detection limit of X-ray system.

Due to comparison between ICP-OES and SEM-EDX analysis, relatively high value of the correlation coefficient was achieved (*R* ^2^ = 0.9891).* R*-squared value close to 1 confirmed both techniques to give the similar results of surface elemental composition and thus to be equally useful in biosorption investigations.

## 4. Conclusions

Biosorption of microelement ions: copper, cobalt, zinc, and manganese, from aqueous solutions by* Spirulina* biomass was investigated to assess the applicability of enriched algae as a feed supplement for livestock. Parallel experiments in single- and multicomponent system were performed. In present work, adsorptive properties of cultivated under laboratory conditions* Spirulina maxima* were verified by comparing to commercially available, lyophilized* Spirulina* sp. Both examined biosorbents were proved to increase. The results obtained from potentiometric titration showed that higher pH value favored metal ions biosorption, since more binding sites were deprotonated. Thereby, ion exchange was confirmed as the dominant mechanism of the process. Although, biosorption capacity was over 3 times higher for* S. maxima* than commercial lyophilisate, loading* Spirulina* sp. with microelement ions increased their surface concentration from 12 up to 1,760 times. The affinity of biomass for microelement cations, determined on the example of pristine and loaded* Spirulina* sp., in single- and multicomponent system was Mn(II) > Cu(II) > Zn(II) > Co(II) and Cu(II) > Mn(II) > Co(II) > Zn(II), respectively. FTIR analysis showed similar chelating characteristics of metal coordination groups to results evaluated with titration. The binding sites present on* Spirulina *cell wall included carboxyl, phosphoryl, and either hydroxyl or amine groups. Sulfonate groups were also proved to participate in ion sorption (FTIR spectra). The concentration of elements on the surface of natural and metal ion-loaded biomass was assessed by both ICP-OES and SEM-EDX techniques. Since relatively high value of correlation coefficient was shown between these systems, usefulness of SEM-EDX analysis in biosorption studies on* Spirulina* biomass was confirmed, as well.

## Figures and Tables

**Table 1:** The composition of the Schlösser medium.

Component	Concentration, g L^−1^
NaHCO_3_	13.61
Na_2_CO_3_	4.03
K_2_HPO_4_	0.5
NaNO_3_	2.5
K_2_SO_4_	1.0
NaCl	1.0
MgSO_4_ *·*7H_2_O	0.2
CaCl_2_	0.03026
FeSO_4_ *·*7H_2_O	0.01
EDTA	0.08

Microelement solution, g L^−1^	

ZnSO_4_ *·*7H_2_O	1.0
MnSO_4_ *·*5H_2_O	2.0
H_3_BO_3_	5.0
Co(NO_3_)_2_ *·*6H_2_O	5.0
Na_2_MoO_4_ *·*2H_2_O	5.0
CuSO_4_ *·*5H_2_O	1.0
FeSO_4_ *·*7H_2_O	0.7
EDTA	0.8
Deionized water	981

**Table 2:** Relative growth rate of continuous cultures in the reactors of different volumes.

Capacity, L	*μ*, day^−1^	*R* ^2^
1	0.145	0.973
5	0.145	0.978
40	0.285	0.987

**Table 3:** Relative growth rates of *Spirulina maxima* cells in culture with biomass reception.

Time interval	*μ*, day^−1^	*R* ^2^
I	0.285	0.987
II	0.0439	0.990
III	0.0503	0.985
IV	0.0464	0.976
V	0.0331	0.949
VI	0.0275	0.876
VII	0.0281	0.923

**Table 4:** Identified functional groups on surface of natural and Cu(II)-loaded *Spirulina* biomass.

Bond	*Spirulina *sp.	*Spirulina maxima *	Cu(II)-*Spirulina maxima *	Component
	Wave number, cm^−1^		
*≣*P–O–, –SO_3_–	861.48	832.89	—	Polysaccharides
*γ* CC/CO, *γ* OH	1049.44 1150.86	1047.54 1149.59	1049.80 1154.21	Polysaccharides, Polysaccharides ethers
*δ* C–O–C, *δ* OH	1241.96	1243.45	1235.52	Amides, Polysaccharides, Esters
*γ*_S_ COO^−^	1400.08	1401.07	1407.45	Carboxyl group
*δ* CH/CH_2_	1454.64	1452.26	1452.12	Alkyl chain
*δ* NH	1541.50	1544.70	1536.32	Secondary amides
*γ* C=O, *γ* CN	1654.16	1657.15	1656.16	Protein primary amides
*γ*_S_ CH_3_	2874.17	2874.50	2874.13	Alkyl chain
*γ*_AS_ CH_3_	2927.79	2927.72	2927.40	Alkyl chain
*γ*_S_ CH_2_	2960.11	2960.13	2959.39	Alkyl chain
*γ* NH_3_	3302.49	3310.08	3372.02	Amines

**Table 5:** Surface functional groups and their concentration evaluated with potentiometric titrations.

Functional group	Microalga(e)			
	*Spirulina maxima *		*Spirulina *sp.	
	pK_*a*_	Concentration meq g^−1^	pK_*a*_	Concentration meq g^−1^
Carboxyl	2.66	2.99	2.77	0.998
Phosphoryl	6.68	1.73	6.96	0.573
Amine or hydroxyl	10.9	3.27	10.8	0.889

Total		7.99		2.46

**Table 6:** Atomic concentration of elements present on the surface of pristine and metal cation-loaded *Spirulina *sp., assessed according to the analysis of X-ray spectrum.

Element	Atomic concentration of elements, % of all detected ions						Detection limits, %
	Natural biomass	Co-*Spirulina *sp.	Cu-*Spirulina *sp.	Mn-*Spirulina *sp.	Zn-*Spirulina *sp.	Co, Cu, Mn, Zn-*Spirulina *sp.	
∗							
C	17.9 ± 4.86	54.8 ± 8.99	44.7 ± 7.94	25.6 ± 2.23	47.9 ± 8.89	33.7 ± 6.81	0.277–100
Cl	1.80 ± 0.12	0.71 ± 0.08	0.42 ± 0.07	1.60 ± 0.16	2.0 ± 0.13	0.20 ± 0.05	0.001–2.621
O	59.31 ± 11.2	39.4 ± 6.76	42.9 ± 7.67	38.3 ± 3.31	42.8 ± 8.24	54.9 ± 10.14	0.525–100
P	0.26 ± 0.06	0.69 ± 0.06	1.37 ± 0.09	1.73 ± 0.13	0.69 ± 0.07	0.29 ± 0.05	0.001–2.013
S	0.49 ± 0.07	0.00 ± 0.00	0.35 ± 0.07	1.37 ± 0.16	0.86 ± 0.09	1.36 ± 0.09	0.001–2.307

∗∗							
Co	0.08 ± 0.08	0.95 ± 0.13	<LLD	<LLD	<LLD	1.16 ± 0.15	0.076–6.924
Cu	0.13 ± 0.11	<LLD	9.27 ± 0.69	<LLD	<LLD	2.85 ± 0.30	0.083–8.040
Fe	0.05 ± 0.06	0.15 ± 0.09	0.15 ± 0.09	<LLD	0.41 ± 0.10	0.36 ± 0.10	0.060–6.398
Mn	0.02 ± 0.00	<LLD	<LLD	27.6 ± 1.27	<LLD	1.74 ± 0.15	0.063–5.894
Zn	<LLD	<LLD	<LLD	<LLD	1.76 ± 0.31	0.18 ± 0.26	0.001–8.630

∗∗∗							
Ca	0.12 ± 0.06	<LLD	<LLD	0.17 ± 0.11	0.19 ± 0.07	<LLD	0.341–3.690
K	2.58 ± 0.18	0.22 ± 0.06	<LLD	0.08 ± 0.09	0.00 ± 0.01	0.28 ± 0.07	0.001–3.312
Na	12.5 ± 0.85	0.42 ± 0.05	<LLD	1.09 ± 0.19	1.01 ± 0.11	1.60 ± 0.13	0.001–1.041

^*^macroelements, ∗∗microelements, ∗∗∗alkali, and alkaline earth metals.

<LLD: lower limit of detection.
